# Diversity of antibiotic resistance genes and staphylococcal cassette chromosome *mec* elements in faecal isolates of coagulase-negative staphylococci from Nigeria

**DOI:** 10.1186/1471-2180-14-106

**Published:** 2014-04-26

**Authors:** Luca Agostino Vitali, Dezemona Petrelli, Adebayo Lamikanra, Manuela Prenna, Ezekiel Olugbenga Akinkunmi

**Affiliations:** 1 School of Pharmacy, University of Camerino, Camerino, Italy; 2 School of Biosciences and Biotechnology, University of Camerino, Camerino, Italy; 3 Department of Pharmaceutics, Faculty of Pharmacy, Obafemi Awolowo University, Ile-Ife, Nigeria

**Keywords:** Coagulase negative staphylococci, Methicillin resistance, Antibiotic resistance genes, Gastrointestinal tract

## Abstract

**Background:**

Coagulase-negative staphylococci (CoNS) are opportunistic pathogens found as colonisers of the human gut. This study was carried out to examine the genetic resistance mechanisms in faecal isolates of CoNS. The study investigated 53 non-duplicate CoNS isolates obtained from the fresh stool samples of apparently healthy subjects in the community of Ile-Ife, South-Western Nigeria. Antibiotic susceptibility testing was assessed by the disc diffusion test while antibiotic resistance genes were analysed by PCR. *mecA* positive isolates were analysed by Staphylococcal Chromosome Cassette *mec* (SCC*mec*) and cassette chromosome recombinase (*ccr*) complex typing methods.

**Results:**

Resistance genes were detected only in isolates that showed resistance by phenotypic screening. The *aac(6′)–aph(2″)* gene was detected in all the three isolates resistant to gentamicin. Four of the five erythromycin resistant isolates were positive for the *ermC* gene*,* the remaining isolate carried the *msrA* gene. The *tetK* gene was detected in 6 of the 7 tetracycline resistant isolates while 4 possessed the *tetM* gene. Three of the isolates (*S. haemolyticus*, *S. xylosus* and *S. capitis*) had both genes. Several SCC*mec* types were found: SCC*mec* I- *ccrAB*β2-α2 (4 isolates: 3 *S. epidermidis*, 1 *S. warneri*), SCC*mec*IVb- *ccrAB*β2-α3 (1 isolate: *S. epidermidis*), SCC*mec*IVd- *ccrAB*β2-α3 (8 isolates: 3 *S. epidermidis*, 2 *S. xylosus*, 1 *S. saprophyticus*, 1 *S. warneri*, 1 *S. capitis*), and untypable (2 isolates: *S. epidermidis*).

**Conclusion:**

This genetic background could be a reservoir for interspecies gene transfer among CoNS and *S. aureus* in the intestinal tract.

## Background

Coagulase-negative staphylococci (CoNS) are opportunistic pathogens commonly associated with nosocomial infections [1]. Most CoNS strains have been reported to have acquired resistance to methicillin and almost all classes of antimicrobial agents [2,3]. The high resistance rates among CoNS have reduced the ability of health care to treat infections associated with them and led to a prolonged course of infections with severe consequences.

In the vast majority of staphylococcal isolates, resistance to macrolides such as erythromycin has been reported to be due to N^6^-dimethylation of a 23S rRNA adenine residue preventing macrolide binding to the 50S ribosomal subunits. In the hospital setting, clinical isolates possessing the *erm*(*A*) and/or *erm(C)* gene coding for rRNA methylases were isolated more frequently than *erm(B)* positive ones [4]. The expression of methylases is usually induced by the presence of 14- or 15-membered macrolides via a translational attenuation mechanism. Modification by mutation of the translation attenuation region may lead to constitutive expression of the methylases even in the absence of inducer macrolides [5]. When expressed, methylases also confer cross-resistance to lincosamides and to streptogramin B compounds (MLS_B_ phenotype). Also point mutations, mapping within the sequence of the domain V of the 23S rRNA or in the proteins of the large ribosomal subunit L4 and L22 may be responsible for MLS_B_ resistance [5]. For full resistance to the streptogramine combination quinupristin-dalfopristin, strains need to carry additional resistance to streptogramin A compounds, which may be mediated by acetylation (acetyl transferase genes *vat(A), vat(B)* and *vat(C)*, or by putative efflux pumps encoded by *vga(A)* and *vga(B)*[5,6].

Tetracycline resistance in staphylococci is either based on the expression of a ribosomal protection factor encoded by the widely disseminated *tet(M)* gene or mediated by *tet(K)* mediated efflux of the antibiotics [7]. For aminoglycoside resistance, the presence of aminoglycoside – modifying enzyme genes *aac(6′)-aph (2″), aph(3′)-IIIa* and *ant(4′)-Ia* has been analysed. The most frequently encountered gene in staphylococci is the *aac(6′)-aph(2″)* which codes for a bifunctional enzyme and confers resistance to gentamicin, tobramycin, kanamycin and when over-expressed to amikacin but not to streptomycin [8]. For the quinolones such as ciprofloxacin and pefloxacin, a main mechanism of resistance is the spontaneous accumulation of mutations in the genes encoding subunits of the DNA gyrase (*gyrA* and *parC*) [9]. Other important antimicrobials include chloramphenicol and co-trimoxazole (trimethoprim + sulphamethoxazole). Resistance to chloramphenicol is mainly mediated by the *catA* gene which is responsible for the chloramphenicol acetyl transferase while co-trimoxazole resistance is due to mutations of the enzyme dihydrofolate reductase encoded by the *dhfr* gene [10].

Methicillin resistance in staphylococci is mainly due to the expression of the *mecA* gene, which specifies penicillin binding protein 2a (PBP2a), a transpeptidase with a low affinity for β-lactams [11]. *mecA* is located on a 21-to 67-kb mobile genetic element (MGE) called Staphylococcal Chromosome Cassette *mec* (SCC*mec*) [11,12].

Different *SCCmec* elements in staphylococci have been classified and characterized according to the combination of two parts: the *ccr* complex and the *mec* complex. Cassette chromosome recombinase (*ccr*) genes (*ccrC* or the pair of *ccrA* and *ccrB*) encode recombinases that mediate integration and excision of SCC*mec* into and from the chromosome [12-14]. The *ccr* gene(s) form the *ccr* gene complex. The *mec* gene complex on the other hand, consists of *mecA*, *mec*R1 and *mec*I regulatory genes and associated insertion sequences and has been classified into six different classes: A, B, C1, C2, D and E [13,14]. The regions located between these complexes are called J (joining) regions. In every SCC*mec* elements there are three of these regions (J1-J3) and polymorphisms in the regions are used for the definition of SCC*mec* type IV subtypes [15]. In addition to *ccr* and *mec* gene complexes and J regions, SCC*mec* contains a few other genes or pseudogenes that does not appear to be essential to the bacterial cell with exceptions including various other MGE, e.g. insertion sequences, transposons and integrated plasmids mediating resistance to non-β-lactam antibiotics or heavy metals [12].

Based on the classes of the *mec* gene complex and the *ccr* gene types, eleven types (I to XI) of SCC*mec* have been assigned for *Staphylococcus aureus*[15,16]. However, only type I-V are globally distributed while others appear to exist as local strains in the country of origin [13,15]. Only the type I-V, have so far been reported in *S. aureus* and type V in two isolates of *S. haemolyticus* from Nigeria [14]. Several SCC*mec* subtypes, subtypes IIA to E and subtypes IVa to IVg and SCC*mec* type V_T_ have been reported in the literature but no report exists from Nigeria as far as we know [13,15].

As methicillin resistance is prevalent in CoNS, methicillin-resistant CoNS (MRCoNS) may serve as a large reservoir of SCC*mec* available for *S. aureus* to form methicillin resistant *S. aureus* (MRSA) [12,16]. Studies have shown that SCC*mec* elements are more diverse in MRCoNS and new *ccr* genes are still being continually identified in various strains of MRCoNS [16].

In this study, in order to examine the genetic drug resistance mechanisms in faecal isolates of CoNS, the presence of antibiotic resistance genes consisting of *mecA, erm(A), erm(B), erm(C), msr(A), tet(M), tet(K)* and *aac(6′)–aph(2″)* and the globally distributed SCC*mec* types and subtypes were analysed by PCR. This is the first report on antibiotic resistance genes of CoNS in Nigeria as well as,to our knowledge, the first report on the SCC*mec* elements in human intestinal CoNS isolates.

## Methods

### Bacterial strains

CoNS isolates were obtained from freshly voided stool samples of apparently healthy children and adult subjects (n = 117) who came for immunizations at five healthcare institutions and households in the community of Ile-Ife, in South-Western Nigeria who gave consent for sample collection. In this study, only staphylococcal isolates were analyzed while clinical data of human subjects were not collected. The study was approved by the Obafemi Awolowo University Postgraduate Research Committee and the Review Boards of the institutions where samples were collected. Parental consent was obtained for each of the children used in the study.

### Isolation and identification of staphylococci

All the isolates were negative to the coagulase slide and tube tests and for the *S. aureus*-specific *nuc* gene as determined by PCR using the protocol previously described [17]. Isolates were further identified to species level by morphological characteristics and by biochemical tests as described previously [2]. Further species differentiation was done by the Vitek 2 apparatus (bioMerieux, Inc. Durham, NC).

### Antibiotic sensitivity test

Antibiotics tested were penicillin V, oxacillin, gentamicin, erythromycin, tetracycline, co-trimoxazole, chloramphenicol, amoxicillin-clavulanate, ciprofloxacin and pefloxacin. The antibiotics screened were selected based on their use in Nigeria. Susceptibility testing was performed by the standard disc diffusion method as approved by the Clinical Laboratory Standard Institute [18].

### Extraction of DNA

Genomic DNA of CoNS isolates were prepared from a 2 mL overnight Tryptone Soy Broth (Oxoid, England) culture using a GenElute™ Bacterial Genomic DNA Kit (Sigma-Aldrich).

### PCR screening of antibiotic resistance determinants

PCRs were perfomed on a Biometra thermocycler (Biometra, USA). All reactions were performed in a 25 μl volume containing: 10 mM Tris/HCl (pH 8.3), 50 mM KCl, 1.25 mM MgCl2, 100 μM each dATP, dCTP, dGTP and dTTP, 1 μM each oligonucleotide primer, 1 U Taq polymerase (Sigma-Aldrich) and 200 ng template DNA. All strains were investigated for the presence of *mecA*[19]; *tet(K)* and *tet(M)*[20]; *erm(A)*, *erm(B)*, *erm(C)*, *msr(A)*[19]; and *aac(6′)–aph(2″)* genes [19]. PCR products were analysed in agarose gel (1.5%) electrophoresis in 1X Tris-borate-EDTA buffer (TBE) at pH 8.3. Electrophoresis was carried out with an appropriate molecular ladder to determine fragment sizes.

### SCC*mec* typing

SCC*mec* typing was performed using the PCR schemes previously published [14,15,20,21]. A single PCR was performed for each gene. For isolates in which SCC*mec* could not be typed, classes of the *mec* gene complex and the *ccr* gene complex (ccrAB1, ccrAB2, ccrAB3 and ccrC1) were examined by additional PCRs using the primers described previously [14]. SCC*mec* types were assigned based on the *mec* complex classes and the *ccr* gene types according to the criteria set for *S. aureus*[14,15].

Positive control strains used in the determination of the SCC*mec* type were the MRSA strains COL/SCC*mec* type I-ST250, BK2464/SCC*mec* type II- ST5, HUSA304 /SCC*mec* type III- ST239, PL72/SCC*mec* type IVh-ST5 and BK2529/SCC*mec* type V-ST8 [17]. As no control strains were available for the remaining SCC*mec* type IV subtypes, we run the simplex PCRs of each using available protocols and correlating the amplicon sizes obtained with those of the literature [15].

## Results

### Carriage of CoNS strains by subjects

From 117 subjects screened, 53 staphylococcal isolates were obtained; in particular *S. epidermidis* (n = 20), *S. haemolyticus* (n = 10), *S. saprophyticus* (n = 5), *S. capitis* (n = 5), *S. lugdunensis* (n = 2), *S. warneri* (n = 4), *S. xylosus* (n = 4), and *S. cohnii* (n = 3).

### Antibiotics susceptibility testing

Resistance rate was generally low in all isolates showing 100.0%, 98.1%, 94.3%, 92.5%, 90.6%, and 86.8% susceptibility to pefloxacin, ciprofloxacin, gentamicin, chloramphenicol, erythromycin, and tetracycline, respectively (Table 1). Higher resistance rate were obtained for amoxicillin-clavulanic acid (58.5%) and co-trimoxazole (35.8%). All the organisms were resistant to Penicillin V. Oxacillin-resistant isolates were 28.3% of total.

**Table 1:** Antibiotic resistance of CoNS isolates from faecal samples

** Antimicrobial**	**Number (%) of resistant isolates**		
	**MRCoNS (n = 15)**	**MSCoNS (n = 38)**	**Total (n = 53)**
Penicillin V (PEN)	15 (100)	38 (100)	53 (100)
Oxacillin (OXA)	15 (100)	0 (0)	15 (28.3)
Gentamicin (GEN)	1 (6.7)	2 (5.3)	3 (5.7)
Erythromycin (ERY)	1 (6.7)	4 (10.5)	5 (9.4)
Tetracycline (TET)	1 (6.7)	6 (15.8)	7 (13.2)
Co-trimoxazole (COT)	14 (93.3)	5 (13.2)	19 (35.8)
Chloramphenicol (CL)	2 (13.3)	2 (5.3)	4 (7.5)
Amoxicillin-clavulanate (AMC)	15 (100)	16 (42.1)	31 (58.5)
Ciprofloxacin (CIP)	1 (6.7)	0 (0)	1 (1.9)
Pefloxacin (PEF)	(0)	0 (0)	0 (0)

### PCR for the detection of antibiotic resistance genes

Correlation between phenotypes and genotypic traits of resistance to the antibiotics was absolute. The *aac(6′)-aph(2″)* gene was detected in all the three isolates resistant to gentamicin while four out of the five erythromycin resistant isolates (2 *S. epidermidis*, 2 *S. haemolyticus*, 1 *S. cohnii*) were positive to *erm(C).* The remaining *S. haemolyticus* isolate had *msr(A)* gene. The *tet(K)* gene was detected in 6 (3 *S. haemolyticus*, 1 *S. xylosus*, 1 *S. capitis*, 1 *S. cohnii*) out of the 7 tetracycline resistant isolates while 4 (2 *S. haemolyticus*, 1 *S. xylosus* and 1 *S. capitis*) possessed the *tet(M)* gene. Three of the isolates (*S. haemolyticus*, *S. xylosus* and *S. capitis*) had both genes. All the fifteen oxacillin resistant isolates possess the *mecA* gene and were taken as MRCoNS.

### SCC*mec* typing

SCC*mec* types were assigned for 13 of the *mecA* positive isolates (Table 2). SCC*mec* type comprised of SCC*mec*I- ccrABβ2-α2 (4 isolates: 3 *S. epidermidis*, 1 *S. warneri*), SCC*mec*IVb- ccrABβ2-α3 (1 isolate: *S. epidermidis*), SCC*mec*IVd- ccrABβ2-α3 (8 isolates: 3 *S. epidermidis*, 2 *S. xylosus*, 1 *S. saprophyticus*, 1 *S. warneri*, 1 *S. capitis*). Two of the *mecA* positive isolates (*S. epidermidis*) were found to be untypable.

**Table 2:** **Phenotypes and genotypes of antibiotic resistance and SCC** ***mec *** **types**

	**Phenotype**								**Genotype**										
**Strain (Unique strain ID)**	**PEN**	**OXA**	**GEN**	**ERY**	**TET**	**COT**	**CL**	**CIP**	***nuc***	***mecA***	***aac-aph***	***erm(A)***	***erm(B)***	***erm(C)***	***msrA***	***tetM***	***tetK***	**SCC*mec *** **type**	**ccr complex**
*S. capitis* (SC01)	R	R	S	S	S	R	S	S	-	+	-	-	-	-	-	-	-	IVd	ccrABβ2-α3
*S. epidermidis* (SE01)	R	R	S	S	S	R	S	S	-	+	-	-	-	-	-	-	-	I	ccrABβ2-α2
*S. epidermidis* (SE02)	R	R	S	S	S	R	S	S	-	+	-	-	-	-	-	-	-	I	
*S. epidermidis* (SE03)	R	R	S	S	S	R	S	S	-	+	-	-	-	-	-	-	-	I	
*S. epidermidis* (SE04)	R	R	S	S	S	R	S	S	-	+	-	-	-	-	-	-	-	IVb	ccrABβ2-α3
*S. epidermidis* (SE05)	R	R	R	S	S	R	R	R	-	+	+	-	-	-	-	-	-	IVd	ccrABβ2-α3
*S. epidermidis* (SE06)	R	R	S	S	S	R	S	S	-	+	-	-	-	-	-	-	-	IVd	ccrABβ2-α3
*S. epidermidis* (SE07)	R	R	S	S	S	R	S	S	-	+	-	-	-	-	-	-	-	IVd	ccrABβ2-α3
*S. epidermidis* (SE08)	R	R	S	S	S	S	S	S	-	+	-	-	-	-	-	-	-	untypable	Untypable
*S. epidermidis* (SE09)	R	R	S	R	S	R	S	S	-	+	-	-	-	+	-	-	-	untypable	Untypable
*S. saprophyticus* (SS01)	R	R	S	S	S	R	S	S	-	+	-	-	-	-	-	-	-	IVd	ccrABβ2-α3
*S. warneri* (SW01)	R	R	S	S	S	R	S	S	-	+	-	-	-	-	-	-	-	I	
*S. warneri* (SW02)	R	R	S	S	S	R	S	S	-	+	-	-	-	-	-	-	-	IVd	ccrABβ2-α3
*S. xylosus* (SX01)	R	R	S	S	R	R	R	S	-	+	-	-	-	-	-	+	+	IVd	ccrABβ2-α3
*S. xylosus* (SX02)	R	R	S	S	S	R	S	S	-	+	-	-	-	-	-	-	-	IVd	ccrABβ2-α3
*S. capitis* (SC02)	R	S	S	S	S	S	S	S	-	-	-	-	-	-	-	-	-		
*S. capitis* (SC03)	R	S	S	S	S	S	S	S	-	-	-	-	-	-	-	-	-		
*S. capitis* (SC04)	R	S	S	S	R	S	S	S	-	-	-	-	-	-	-	+	+		
*S. capitis* (SC05)	R	S	R	S	S	S	S	S	-	-	+	-	-	-	-	-	-		
*S. cohnii* (SCO01)	R	S	S	S	S	S	S	S	-	-	-	-	-	-	-	-	-		
*S. cohnii* (SCO02)	R	S	S	R	R	S	R	S	-	-	-	-	-	+	-	-	+		
*S. cohnii* (SCO03)	R	S	S	S	S	S	S	S	-	-	-	-	-	-	-	-	-		
*S. epidermidis* (SE10)	R	S	R	S	S	S	S	S	-	-	+	-	-	-	-	-	-		
*S. epidermidis* (SE11)	R	S	S	S	S	S	S	S	-	-	-	-	-	-	-	-	-		
*S. epidermidis* (SE12)	R	S	S	S	S	R	S	S	-	-	-	-	-	-	-	-	-		
*S. epidermidis* (SE13)	R	S	S	S	S	S	S	S	-	-	-	-	-	-	-	-	-		
*S. epidermidis* (SE14)	R	S	S	S	S	S	S	S	-	-	-	-	-	-	-	-	-		
*S. epidermidis* (SE15)	R	S	S	S	S	S	S	S	-	-	-	-	-	-	-	-	-		
*S. epidermidis* (SE16)	R	S	S	R	R	R	S	S	-	-	-	-	-	+	-	-	-		
*S. epidermidis* (SE17)	R	S	S	S	S	S	S	S	-	-	-	-	-	-	-	-	-		
*S. epidermidis* (SE18)	R	S	S	S	S	S	S	S	-	-	-	-	-	-	-	-	-		
*S. epidermidis* (SE19)	R	S	S	S	S	S	S	S	-	-	-	-	-	-	-	-			
*S. epidermidis* (SE20)	R	S	S	S	S	S	S	S	-	-	-	-	-	-	-	-	-		
*S. haemolyticus* (SH01)	R	S	S	S	S	S	S	S	-	-	-	-	-	-	-	-	-		
*S. haemolyticus* (SH02)	R	S	S	S	S	S	S	S	-	-	-	-	-	-	-	-	-		
*S. haemolyticus* (SH03)	R	S	S	S	S	R	S	S	-	-	-	-	-	-	-	-	+		
*S. haemolyticus* (SH04)	R	S	S	S	R	S	R	S	-	-	-	-	-	-	-	-	+		
*S. haemolyticus* (SH05)	R	S	S	S	R	S	S	S	-	-	-	-	-	-	-	+	+		
*S. haemolyticus* (SH06)	R	S	S	R	R	R	S	S	-	-	-	-	-	-	+	+	-		
*S. haemolitucus* (SH07)	R	S	S	R	S	R	S	S	-	-	-	-	-	+	-	-	-		
*S. haemolitycus* (SH08)	R	S	S	S	S	S	S	S	-	-	-	-	-	-	-	-	-		
*S haemolyticus* (SH09)	R	S	S	S	S	S	S	S	-	-	-	-	-	-	-	-	-		
*S. haemolyticus* (SH10)	R	S	S	S	S	S	S	S	-	-	-	-	-	-	-	-	-		
*S. lugdunensis* (SL01)	R	S	S	S	S	S	S	S	-	-	-	-	-	-	-	-	-		
*S. lugdunensis* (SL02)	R	S	S	S	S	S	S	S	-	-	-	-	-	-	-	-	-		
*S. saprophyticus* (SS02)	R	S	S	S	S	S	S	S	-	-	-	-	-	-	-	-	-		
*S. saprophyticus* (SS03)	R	S	S	S	S	S	S	S	-	-	-	-	-	-	-	-	-		
*S. saprophyticus* (SS04)	R	S	S	S	S	S	S	S	-	-	-	-	-	-	-	-	-		
*S. saprophyticus* (SS05)	R	S	S	S	S	S	S	S	-	-	-	-	-	-	-	-	-		
*S. warneri* (SW03)	R	S	S	S	S	S	S	S	-	-	-	-	-	-	-	-	-		
*S. warneri* (SW04)	R	S	S	S	S	S	S	S	-	-	-	-	-	-	-	-	-		
*S. xylosus* (SX03)	R	S	S	S	S	S	S	S	-	-	-	-	-	-	-	-	-		
*S. xylosus* (SX04)	R	S	S	S	S	S	S	S	-	-	-	-	-	-	-	-	-		
*Summary*	R=53	R=15	R=3	R=5	R=7	R=19	R=4	R=1	+=0	+=15	+=3	+=0	+=0	+=4	+=1	+=4	+=6		

R, Resistant; S, susceptible; +, positive in specific PCR; −, negative in specific PCR.

## Discussion

Coagulase-negative staphylococci (CoNS) isolates from various sources have been identified as reservoir of genetic determinants of antibiotic resistance such as antibiotic resistance genes and various SCC*mec* elements [16]. The horizontal transfer of these resistance genes is thought to contribute to the reported increasing rate of resistance in strains of these organisms as well as in *S. aureus*. In the same vein, the horizontal transfer of SCC*mec* elements has been thought to contribute to the generation of new strains of methicillin resistant staphylococci, including MRSA. One of the environments which favour such an exchange is the gastrointestinal tract [22] and this study thus focused on the presence of these genes in CoNS present in the gut and isolated from stool samples of healthy individuals from Nigeria.

Different rates of resistance were recorded for the various antibiotics tested and full correlation between phenotypes and genotypic traits of resistance to the antibiotics was found. Erythromycin resistance in staphylococci has been reported to be predominantly mediated by erythromycin-resistant methylase encoded by the *erm* genes [6,23], namely *erm(A)*, *erm(B)* and *erm(C). erm(A)* is found on the transposon Tn*554* with a single specific site for insertion into the *S. aureus* chromosome while *erm(B)* gene is found on the transposon Tn*551* of a penicillinase plasmid. The *erm(C)* gene on the other hand is responsible for constitutive or inducible resistance to erythromycin and is generally located on small plasmids [5,6,23]. This indicates the high capacity of these genes to be horizontally transferred to recipient strains. Our study showed that 4 (*S. epidermidis* 2, *S. haemolyticus* 1, *S. cohnii* 1) out of 5 erythromycin resistant isolates possessed *erm(C)* genes. The *erm(A)* and *erm(B)* genes were absent. Studies conducted in other countries such as Italy, Denmark, and Tunisia also reported *erm(C)* as the prevalent gene in clinical isolates of erythromycin resistant *S. epidermidis*[7,23,24]. One of the erythromycin resistant *S. haemolyticus* strain was found to possess the *msr(A)* gene which encodes an ATP-dependent efflux pump conferring resistance to 14- and 15-membered macrolides [5]. Six of the tetracycline resistant strains (3 *S. haemolyticus*, 1 *S. capitis*, 1 *S. xylosus*, and one *S. cohnii* ) were also found to possess the *tet(K)* gene which encodes for an efflux mechanism of resistance. The presence of these efflux pumps in the CoNS strains from stool samples may contribute to the increase in incidence of resistance to other antimicrobial agents that are targeted by these efflux pumps, such as some antiseptics and disinfectants. The overall prevalence of tetracycline resistance is noteworthy and may reflect the overuse of different tetracyclines in the study area. Despite the fact that tetracycline is not officially recommended for children in the study area, tetracycline capsules are widely available in all stores in Nigeria and it is one of the most used drugs in this country. On the other hand, co-trimoxazole was the first line oral antibiotic recommended by World Health Organisation’s Integrated Management of Childhood Illnesses (IMCI) for the treatment of local bacterial infection in the infant and thus it is widely prescribed by many physicians and is often used as a prophylaxis in many diseases in the study area. Hence the high resistance rate obtained for it may not be out of place. The same applies to amoxicillin-clavulanate which are often prescribed instead of β-lactamase susceptible penicillins in the study area.

It appears that the incidence of CoNS isolates from faeces carrying aminoglycosides, macrolides, and tetracycline resistant genes may be due to the transfer of resistance genes inter- and intra species thereby contributing to the dissemination of staphylococcal resistance. The association between methicillin resistance and resistance to antibiotics belonging to classes other than beta-lactam is of particular interests. For instance the set of MRCoNS included in this study presents some examples. Strain SEO5 is resistant to aminoglycosides and is positive to SCC*mec*IVd, the structure of which is known to be lacking genetic determinants responsible for resistance to aminoglycosides. Conversely SCC*mec* type II and IVc carry within them pUB110 and Tn4001, respectively. By comparison to other *S. epidermidis* within the MSCoNS subgroup, it can be concluded that the element carrying the aminoglycoside resistance gene is outside the SCC*mec* (see strain SE10). Of note is the isolation of strains possessing a pattern of multi-resistance (e.g. SE05 and SX01). This finding is interesting as samples were isolated from healthy people. Multi-resistance is more often recorded in the hospital settings and in the case of staphylococci, is associated with the use of medical devices such as catheters (25). This information is important for the control of nosocomial infections and confirms the importance of CoNS as a reservoir of resistance determinants. In addition to this, given the extensive use of these antibiotics in the study area, the widespread occurrence of resistance mechanisms with potential for rapid dissemination necessitates the implementation of surveillance programmes to monitor the development and spread of antimicrobial resistance in our country.

In agreement with previous studies [16,25], the SCC*mec* elements identified in the MRCoNS strains investigated herein exhibited some genetic diversity. Previous reports have indicated that type VI, VII, IX, X and XI are yet to be reported in MRCoNS and type I and VIII are still rare while type II, III, IV and V were more common [11,16,25,26]. Our results are in general agreement with these reports. However, in contrast to an earlier report [25] which found SCC*mec*III as the most common SCC*mec* element (39.3%) followed by SCC*mec*V (36.9%) and SCC*mec*IV (20.2%), our results indicated that SCC*mec*IVd was the most prevalent (53.3%) followed by SCC*mec*I (26.7%) and SCC*mec*IVb (6.7%). It had been suggested that the variations in the distribution of different types of SCC*mec* in MRCoNS depend on the host species and on the geographical locations [25,26]. Our results indicated that most of the type IVd strains isolates were *S. epidermidis* whereas a study conducted in the Netherland reported a prevalence of type IVc in *S. epidermidis* and other staphylococci isolated from pigs [16]. Other studies have found type V SCC*mec* associated with *S. haemolyticus*[16,27]. The higher prevalence of SCC*mec*IV in our strains could be explained by the fact that all the samples in our study were from the community, where this SCC*mec-*type is diffused, whereas the type I, II and III SCC*mec* are more frequently isolated in hospital settings. There is also a variation in the distribution and prevalence of the various SCC*mec* types in MRCoNS in different countries [26]. SCC*mec* type III has been found to be the most prevalent in southern Brazil (52%), SCC*mec* type IV in the United Kingdom (36%), type IVa in Japan (40.8%), and type II in China. Some authors have recently reported type V and untypable elements in two *S. haemolyticus* isolates from Nigeria [27]. Our data add on to this latter study providing information for CoNS other than *S. haemolyticus* circulating in Nigeria.

SCC*mec* could not be classified in two of the MRCoNS isolates. They may belong to other SCC*mec* types not considered in the present investigation or may be among those that cannot be assigned to by currently-available PCR-based methods. Nevertheless the design and validation of a comprehensive SCC*mec* typing classification scheme for MRCoNS is heavily challenged by the frequent isolation of strains possessing “non-typeable” elements or even positive to more than one SCC*mec*-type [16,25]. In our study, SCC*mec* types were assigned by PCR protocols originally developed for SCC*mec* in MRSA [14,15], supporting the general conclusion that the scheme is still suitable as a first screening of SCC*mec* types in MRCoNS. Our results also indicate a large diversity in the J1 region in type IV of SCC*mec* and a large diversity and heterogenous reservoir of SCC*mec* among the MRCoNS isolated from faecal samples of humans. This may be a risk for interspecies horizontal transfer of new SCC*mec* types between CoNS and *S. aureus*[28]. The hypothesis of the particular case of SCC*mec* transfer between *S. epidermidis* and *S. aureus* has also been reported [11]. Although direct proof of transfer was not obtained in this study, SCC*mec* type IVd was present in 8 MRCoNS of various species indicating the possibility of interspecies transfer of SCC*mec* elements in CoNS strains in the gastrointestinal tracts.

## Conclusion

In conclusion, our study indicated that CoNS colonising the gastrointestinal tracts of healthy individuals may represent a reservoir of different antibiotic resistance genes and SCC*mec* elements.

## Abbreviations

CoNS: Coagulase-negative staphylococci; SCCmec: Staphylococcal chromosome cassette *mec*; ccr: Cassette chromosome recombinase; MRSA: Methicillin resistant *S. aureus*; MRCoNS: Methicillin-resistant CoNS.

## Competing interests

The authors declared that they have no competing interest.

## Authors’ contributions

AL, EOA and LAV conceived of the study and participated in its design. AL and LAV participated in the coordination and helped to draft the manuscript. EOA carried out the phenotypic and molecular characterization of the isolates and drafted the manuscript. LAV and DP participated in the molecular genetic studies. MP participated in the co-ordination of the study. All authors read and approved the final manuscript.
